# The Role of Spirituality in Coping with the Psychological Impact of the COVID-19 Pandemic: A South African Case Study

**DOI:** 10.1007/s10943-026-02720-4

**Published:** 2026-06-27

**Authors:** Kevisha Samlal, Naiema Taliep, Ghouwa Ismail

**Affiliations:** 1 Department of Psychology, University of South Africa, Pretoria, South Africa; 2 Institute for Social and Health Sciences, University of South Africa, Lenasia, Johannesburg, South Africa; 3 South African Medical Research Council, Masculinity and Health Research Unit, University of South Africa, Cape Town, Parow, South Africa

**Keywords:** Coping mechanisms, COVID-19, Spirituality, Religion, Grief, Qualitative

## Abstract

The COVID-19 pandemic significantly increased mortality rates and exposed individuals to profound psychological challenges, particularly those related to loss, grief, and death. This qualitative interpretive study explored how spirituality and religion influenced coping mechanisms during the pandemic in Sandton, South Africa. Semi-structured interviews were conducted with ten purposively selected participants (*N* = 10). The findings indicate that spirituality and religious beliefs played a central role in coping with emotional distress, with faith-based practices such as prayer, rituals, and meaning-making fostering resilience under restrictive public health conditions. The study highlights the importance of integrating spiritual resources into mental health interventions during crises.

## Introduction

Throughout history, humanity has faced several pandemics, with COVID-19 being the most recent global pandemic (Liu et al., 2020). Unlike localized epidemics, pandemics spread widely, affecting large populations (Robinson, 2024). In 2020, the World Health Organization (WHO) officially declared COVID-19 a pandemic due to its severity and rapid transmission (World Health Organization, 2023). As of 13 April 2024, the Worldometer Coronavirus Tracker reported 704,753,890 confirmed COVID-19 cases and 7,010,681 reported deaths globally (Worldometer, 2024). The toll of COVID-19 has been immense, with millions of lives lost worldwide (World Health Organization, 2023). Prabhu and Gergen (2021) suggest an actual death toll of approximately 17 million, positioning COVID-19 among the deadliest recorded pandemics.

South Africa promptly took decisive action to halt the spread of the virus and implemented a range of public health and protective measures, including mandatory mask-wearing in indoor public spaces, social distancing, sanitising, and entry requirements at borders (Centers for Disease Control & Prevention, 2021). The country also instituted various alert levels, with Level 5 being the strictest, involving a complete nationwide lockdown to safeguard the well-being of its population (South African Government, 2022; Stiegler & Bouchard, 2020). This rapid response led to the closure of schools and businesses, and to the prohibition of all public gatherings (Omary et al., 2020). Consequently, South Africans’ daily lives, work, and interactions were significantly altered (Stiegler & Bouchard, 2020). The impact of these lockdown measures, particularly restrictions on movement and economic activity, placed a significant burden on individuals, families, and communities both economically and psychosocially (Swart et al., 2022). Shock, uncertainty, and fear were common responses during the early stages of the COVID-19 pandemic.

The pandemic had a profound global impact, including in South Africa, resulting in substantial experiences of loss and grief beyond mortality alone. As the pandemic spread across continents, it created widespread fear, worry, and uncertainty (Saladino et al., 2020). Goveas and Shear (2020) note that many individuals were directly or indirectly affected and experienced various forms of psychological distress, including grief and bereavement. In this context, loss, grief, and death took on significant meaning. Loss extended beyond the death of loved ones to include job and income loss, as well as disruption to daily routines (Closson et al., 2024). Grief was often compounded by the restrictions imposed during the pandemic (Aten, 2021). The finality of death, often experienced in isolation due to social distancing measures, added further complexity to the grieving process. Close family and friends of those who died from COVID-19 also experienced stigma, including social avoidance and rejection (CDC, 2021), largely driven by fear of infection. Other losses included reduced access to support services and significant lifestyle changes (Mosavel et al., 2022). Government restrictions also led to the closure of places of worship, raising concerns about supporting individuals who rely on spiritual and religious practices as coping mechanisms (Mosavel et al., 2022).

Spiritual and religious practices often support mental and emotional well-being (National Alliance on Mental Illness, 2016). This is particularly relevant in South Africa, a multicultural society in which spirituality and religion are integral to daily life. Approximately 80% of the population identifies as Christian, while 15% report no religious affiliation (South African Embassy in the Netherlands, 2026). The remaining population follows various other faiths, including Islam, Hinduism, and Judaism. The South African Constitution supports this diversity and guarantees freedom of religion.

The closure of social, religious, and spiritual gathering spaces during the pandemic created a void in many individuals’ lives, particularly during a period characterized by loss and grief. Mourning during the pandemic was extremely challenging, as individuals received limited face-to-face support and often grieved in isolation. The inability to perform traditional funeral rites contributed to prolonged grief and negatively affected overall well-being (Hamid & Jahangir, 2022). Research has shown that restrictions on mourning rituals may have contributed to complicated grief and persistent complex bereavement disorder (Diolaiuti et al., 2021).

Understanding the psychological challenges associated with loss, grief, and death is therefore essential in informing effective support interventions and coping strategies. Belief systems provide individuals with a framework for understanding the world and navigating adversity (NAMI, 2016). Spirituality and religion offer comfort, meaning, and social support through shared beliefs and practices (Coppola et al., 2021). Evidence suggests that spirituality positively influences well-being, healthcare experiences, and coping outcomes, particularly in South African contexts (Arrey et al., 2016). Spirituality has also been identified as a key coping strategy during stressful situations, promoting resilience and psychological adjustment (Chirico, 2021; Ungureanu & Sandberg, 2010).

Accordingly, this study aimed to explore how participants utilised spiritual and religious resources to cope with the psychological impact of loss, grief, and death during the COVID-19 pandemic. The study examined participants’ experiences of loss, their perspectives on life and death, and the role of spirituality and religion in shaping coping mechanisms during this period of crisis. By focusing on lived experiences, the study provides insights into how religiosity and spirituality informed meaning-making during a global event that significantly altered everyday life.

### Theoretical Framework

The theoretical framework of the study was grounded in the Kübler-Ross model, introduced by psychiatrist Elisabeth Kübler-Ross in 1969. This model provides valuable insights into navigating significant life transitions, including loss, grief, and death, by identifying five stages: denial, anger, bargaining, depression, and acceptance. The Kübler-Ross model offers a structured framework for understanding the emotional processes individuals undergo during major life disruptions, such as those triggered by the COVID-19 pandemic. By examining these stages, researchers can better understand how individuals emotionally respond to and cope with critical life events.

The model is particularly suited to this study because it directly addresses emotional responses to loss and bereavement—the central focus of this research (Kübler-Ross, 1969). Furthermore, its stages provide a conceptual framework for interpreting the coping mechanisms individuals employ, including the use of spiritual and religious resources.

## Methods

### Study Approach and Design

This study employed a qualitative interpretive research design, which facilitated an exploratory approach to understanding the role of spirituality and religion in managing the psychological consequences of loss, grief, and death during the COVID-19 pandemic, as experienced through participants’ perspectives and lived experiences (Dudovskiy, 2016a, 2016b).

In essence, this study integrates an interpretive research framework with the Kübler-Ross model to examine emotional experiences related to life transitions. This combination enables a comprehensive and nuanced understanding of the role of spirituality and religion in coping with the psychological impact of loss, grief, and death during the pandemic.

Additionally, a purposive sampling strategy was employed to recruit participants with direct lived experiences of loss and grief during the pandemic, ensuring depth and relevance of the data collected (Creswell & Poth, 2018). Data was analysed using thematic analysis, allowing for the identification of key patterns and meanings related to spirituality and religious coping within participants’ narratives (Braun & Clarke, 2006).

### Study Setting, Sampling, and Participants

The study was conducted in Sandton, an affluent business and residential suburb within the Johannesburg Metropolitan Municipality, South Africa. As one of the country’s leading economic hubs, Sandton is characterised by modern infrastructure and a diverse population. Its affluence provides access to a wide range of resources, including healthcare, psychological support, and religious services, making it a suitable setting to explore coping mechanisms during the COVID-19 pandemic.

Sandton is also home to professionals across sectors such as health, finance, and education—industries significantly affected by the pandemic, thereby offering diverse perspectives on experiences of loss and resilience. Additionally, the area's religious and cultural diversity enriched the study by allowing for the inclusion of participants from diverse faith backgrounds. The selection of Sandton was both strategic and convenient, as the researcher had proximity to the area, facilitating data collection during COVID-19 restrictions. A purposive sampling strategy was used to select participants who could provide in-depth insights into the research topic.

Table 1*** presents the demographic characteristics of the study participants (N* = *10).*** It provides an overview of key attributes such as age, gender, and relevant background variables, offering contextual insight into the sample and supporting the interpretation of the study findings.

**Table 1:** Participant demographic characteristics (*N* = 10)

Participant	Age	Gender	Employment	Religion	Education level
Participant 1	49	Male	Claims reworks specialist	African traditional and christianity	Grade 12
Participant 2	50	Female	Professional assistant	Judaism	Diploma
Participant 3	30	Female	Medical professional	Islam	Postgraduate Degree (Doctorate)
Participant 4	31	Female	Health claims specialist	Hinduism	Undergraduate degree
Participant 5	35	Male	Team leader coach	Christianity and spiritual	Undergraduate degree
Participant 6	44	Male	Analyst	Christianity	Undergraduate degree
Participant 7	39	Female	Senior training team leader	Christianity	Postgraduate degree
Participant 8	31	Female	Health claims specialist	Hinduism	Grade 12
Participant 9	33	Female	Auditor	Christianity	Postgraduate degree
Participant 10	47	Male	Claims exceptions specialist	African traditional and christianity	Grade 12

Table 1 provides an overview of the participants’ demographic characteristics. All participants were between the ages of 30 and 55 years, with the majority being female (*n* = 6). Three participants completed Grade 12, while the remaining participants (*n* = 7) had tertiary qualifications. Of these, three held postgraduate degrees, three held undergraduate degrees, and one held a diploma.

Most participants identified as Christian (60%), with 20% also incorporating traditional African beliefs. Other religious affiliations included Hinduism (*n* = 2), Islam (*n* = 1), and Judaism (*n* = 1). Although the sample was skewed toward Christianity, this distribution reflects the broader religious demographics of South Africa (South African Embassy in the Netherlands, 2026).

Participants provided rich and reflective accounts of their experiences during the COVID-19 pandemic, including their awareness of the crisis, coping with lockdown restrictions, experiences of loss and grief, and the use of personal and spiritual resources.

### Data Collection Methods and Procedure

This study used semi-structured individual interviews, which are conversational in nature and include open-ended questions (Mouton, 2001), to explore participants’ perceptions and experiences during the pandemic, and how these experiences psychologically affected them in terms of life, loss, grief, and death. This approach also enabled the exploration of the coping strategies participants adopted. The use of semi-structured interviews enabled in-depth discussions and rich data collection.

Participants provided signed informed consent prior to participation, and privacy was maintained during interviews conducted either in person at the workplace or via Microsoft Teams. All interviews were recorded and transcribed, and data were securely stored. Confidentiality was maintained in accordance with the Protection of Personal Information Act (POPIA), with all personal identifiers removed and replaced with pseudonyms. Recordings were securely stored and accessible only to the research team.

Participants were informed that their participation was voluntary and that they could withdraw at any stage without any negative consequences. These measures ensured a safe and respectful environment for participants to share their experiences. Additionally, a clinical psychologist was available to provide support if required (Busetto et al., 2020; Melville & Hincks, 2016).

### Data Analysis

This study employed a thematic analysis approach, a qualitative method used to identify, analyse, and report patterns within data (Caulfield, 2019). The interview transcripts were systematically examined to identify recurring themes and patterns across participants’ accounts. Since the authors ascribe to different religious denominations. Reflexivity was maintained throughout the analytic process to enhance rigour and minimize the influence of researcher subjectivity and potential bias.

The analysis followed several stages, including familiarization with the data, generation of initial codes, identification of themes, review of themes, and final definition and naming of themes (Anderson, 2007; Braun & Clarke, 2006). The findings are presented through a hierarchy of superordinate and subordinate themes that emerged from the analysis.

Guided by a qualitative interpretive design and the Kübler-Ross model, the study explored participants’ subjective experiences of life, loss, grief, and death during the COVID-19 pandemic. The analysis examined psychological coping mechanisms, including spiritual and religious strategies, used to navigate the challenges associated with pandemic-related restrictions. Particular attention was given to the meanings participants ascribed to life and death and the role of spirituality in managing loss and grief.

### Findings and Discussion

Participants from diverse monotheistic and Indigenous belief systems were purposively selected for this study. The research explored psychological coping mechanisms, including the use of spiritual and religious resources, employed by participants to navigate the effects of COVID-19-related restrictions and associated experiences of loss and grief. Table 2*provides a summary of the superordinate themes aligned with the research objectives, a summary of each theme's content, and the key subordinate themes that emerged from the data*.

**Table 2:** Core themes extracted from thematic analysis

Superordinate themes	Summary of theme	Subthemes
Diverse meanings of life and death	This theme encompasses the wide range of interpretations, beliefs, philosophies, and perspectives that individuals and cultures attribute to the concepts of life and death	***General view of life and death*** ***Culture and meanings of life and death***
The impact of government restrictions, loss, grief, and death during COVID-19	This theme explores the participant’s journey, using the Kübler-Ross model of perspectives of loss, grief, and death and changes during the COVID-19 pandemic, including responses to the lockdown and family, home, personal, and work-related matters faced during this time	***Impact of the suddenness of the restrictions*** ***Lockdown and its implications*** ***Loss, death, and grief during the pandemic***
Spiritual and religious coping mechanisms used to navigate loss, death, and grief	This theme focuses on the role of resources in participants’ experiences. This ranges from various resources, including personal, spiritual, and religious, which participants drew on to cope during the pandemic and when faced with challenges such as loss, grief, and death	

Table 2*** presents the core themes identified from the thematic analysis.*** These themes summarise key patterns in participants’ experiences of loss, grief, and spirituality during the COVID-19 pandemic.

As depicted in Table 2, the findings are presented under the following superordinate themes: (1) Diverse meanings of life and death; (2) Impact of government restrictions, loss, and death during COVID-19; and (3) Participants’ coping mechanisms used during the pandemic.

## Diverse Meanings of Life and Death

Participants expressed varied interpretations of life and death, shaped by cultural, spiritual, and personal beliefs. Many engaged in existential reflection, attempting to make sense of mortality during the pandemic. These reflections align with the literature, indicating that individuals construct meaning through their belief systems (Bautista et al., 2017; Krok, 2015). For example, interpretations of death as a transition rather than an endpoint were common, particularly within religious contexts.

Islamic perspectives emphasised the afterlife and divine judgment, aligning with religious teachings (Khan, 1996; Kristiansen & Sheikh, 2012). Similarly, Jewish and Christian beliefs highlighted moral living and spiritual continuity. Research also suggests that even individuals with more secular orientations may still conceptualise forms of continuity after death (Haimila & Muraja, 2023). However, such beliefs may coexist with feelings of uncertainty and existential fear (Haimila & Muraja, 2023; Toscani et al., 2003). Within Hindu traditions, beliefs in reincarnation and karma provide a cyclical understanding of life and death (Highland, 2023). These perspectives reinforce the role of belief systems in shaping interpretations of mortality. The diverse meaning of life and death is presented under two sub-themes: (1) general view of life and death, and (2) culture and meaning of life and death.

### General View of Life and Death

This subtheme considers that participants may have unique interpretations of the meanings of life and death, based on their personal experiences, values, and worldviews, and that this diversity exists. Some participants particularly grappled with existential questions and sought to make sense of life and death, as demonstrated in the following excerpt:

So, for me, I don't know when I die… I, don’t… know, I'm back and forth because of my whole rethinking, my religion and spirituality, and that I'm back and forth about what really happens to us when we die, can we? Umm, can we really, you know, see our loved ones who are behind? Will we see them again in the afterlife like that? But for myself, I don't think I, you know, I haven't thought about death… For me, it's more about … how it will affect the people around me. (Participant 7)

While this participant expressed some sort of ambivalence or uncertainty about death, the excerpt alludes to concern for those who are left behind when the participant dies. Participant 7, who identified as a Christian, noted that she was not a practicing Christian in terms of formal religious rituals and customs, but she nevertheless believes in God, and her view on life is one where she does not see negative life experiences as being a “punishment” or “lesson” from God, stating:

It's more just, that, it's not that I lost my faith, but I have had a bit of a speed bump because I don't know, just experiences in that where I, you know, you question things and trying to figure out where I sit with things because a lot of the things that you're taught… So, my religion is uh, I'm Christian, born and raised… It's just, you know, believing in like God in general, whether it is the God for Christians, Muslims, Jews, or Hindu, you know, just that I think that with the way that we do religion in the world sometimes leads to unnecessary pain and wars and stuff… So, I'm in, in between soul-searching kind of phase … Believing blindly, you know when people were saying things like … when someone's got cancer, it's like, oh, God's trying to teach you a lesson, like, well, that's not good... So, I would never give my child the disease to give to teach them a lesson. (Participant 7)

This extract demonstrates how the participant was grappling with religion and views of the divine, which is a core dimension, one of seven conceptions and beliefs that people hold regarding life, as proposed by Krok (2015), having positive optimistic beliefs about one relationship with the divine as far as religion is concerned. Such existential contemplations or questions are frequently prompted by human beings’ encounters with a variety of personal, environmental, and socio-cultural events and processes (Bautista et al., 2017). According to Krok (2015), individuals can find meaning in life when their experiences convey significant and understandable information or when their lives make sense.

This meaning entails comprehension, which shows humans' capacity to understand and make sense of their lives, including themselves, the external world, and their role in it. In the Islamic faith, death is also perceived as a passage to the afterlife, where individuals are judged according to their faith and actions. As demonstrated in the following quote, Muslims hold the belief in resurrection and the Day of Judgment, where the virtuous are granted entry into paradise while the unrighteous face retribution in hell. A participant explained her view:

My personal view is like my (sic) religious beliefs, [i.e. Islam]. I believe that basically, everybody has an assigned time, so we [are] all meant to go. We believe in life after death, so we will be brought up at a certain point on the day of judgment and be questioned for our deeds. That's why we need to be conscious of what we do and how not to harm people, because everything has its time and we believe that after you get judged on the day of judgment, there's a further and continuous life and that's either hell or heaven and eternal life. (Participant 3)

This view aligns with the instructions of Prophet Muhammad to all Muslims to live in the world as though they were strangers or travellers, emphasizing the transient nature of earthly existence (Khan, 1996). In this context, death signifies a new beginning rather than an end. Muslims believe that life and death are per the will of Allah (God), with everyone’s lifespan predetermined. Death represents the transition to the Hereafter, which is considered the ultimate destination (Kristiansen & Sheikh, 2012). Similar to Muslims and Christians, Jews also believe in an afterlife described as a spiritual realm where souls are believed to go after death, and they also believe in the resurrection of the dead, where the righteous will go, and a realm where the wicked will dwell.

Overall, Jewish beliefs on life after death emphasize the importance of leading a righteous and ethical life, with the hope of being rewarded in the afterlife, as expressed by a participant:

I'm a Jewish person. I grew up in a kosher home and we were observant, not religious. So, I want to go quickly [i.e. die quickly], I don't want to say I don't want like it's uh, it's weird and like I want to go quick for me, and I want to go quick for my family. I don't want there to be suffering… If I'm going to go six feet under, right, but it's the reality is you don't want to die younger before you're all (sic) before your time kind of thing. If you can live longer and please God, it's a good life. (Participant 2)

The same participant explained the various rituals that accompany death and grieving, stating:

… So, the concept is we bury it [the deceased] as soon as possible. So, they can move up to heaven straight away, so … their soul will move into a better place… We say certain prayers on certain Jewish holidays… and, we have the day of remembrance of the Hebrew date of when they passed away and it's called Yahrzeit. So, you, it is the prayers that we do [on] the seven holidays, and your remembrance is the anniversary of their death. (Participant 2)

These perspectives on life and death, particularly the belief in the eternal nature of the soul within Christian, Islamic, and Jewish traditions, are consistent with existing literature. Haimila and Muraja (2023) similarly explored after-death beliefs among science-oriented Finns. While the majority in their study endorsed secular or annihilationist views of death, some participants expressed beliefs in forms of continuation, often framed through enduring social bonds or integration with nature. This suggests that even within secular contexts, notions of continuity after death may persist in symbolic or relational forms, aligning in part with the spiritual continuities emphasized in Abrahamic faiths.

The findings suggest a diversity of beliefs about after-death experiences, even among those who endorse science. They note that it can evoke feelings of dread and fear, and its impact is anticipated to be profound for those who undergo it (Haimila & Muraja, 2023**; **Toscani et al., 2003). Bautista et al. (2017), citing Bauman (2007), argue that individuals strive to reconcile with the inevitability of death, often turning to cultural constructs for solace. They note that among these constructs, the notion that death is not a finality, but a transition from one world to another, stands out prominently. In this perspective, the deceased do not depart from the existing world to dissolve into oblivion; rather, they embark on a journey to an alternate realm, where they persist in existence albeit in a transformed state (Bautista et al., 2017).

Religious systems offer a comprehensive framework of beliefs, perspectives on existence, objectives, and interpretations that aid individuals in understanding the complexities of the world and navigating personal challenges and dilemmas (Krok, 2015). Hindus, for example, believe in reincarnation, which is a cycle of birth, death, and rebirth governed by the law of karma. The goal of Hinduism is to achieve liberation (moksha) from the cycle of rebirth and attain union with the divine (Brahman) (Highland, 2023). One participant explained this cycle as follows:

We believe that we get reincarnated like you either become… as you can be like an animal or person and … like after the person passes away, they spread it out that powder. [I] don’t know what it is. It's not the ashes, but they spread it out and do not use that room for that entire night. And this is in the house of the person who passed away. And the next morning, you see a print, like it looks like, almost like a baby's foot or even like a bird's foot or something. So, we believe in reincarnation. (Participant 4).

The preceding views demonstrate the existence of distinct constitutive features of life and death, life after death, and the destiny of the righteous versus the evil. Awareness of such differences may assist palliative care professionals in meeting the actual needs of terminal patients (Toscani et al., 2003).

### Culture and Meanings of Life and Death

Cultural beliefs played a significant role in shaping participants’ understanding of death. In African traditional contexts, death is viewed as a transition to another realm rather than an end (Obamwonyi, 2016; Rugonye & Bukaliya, 2016). Participants described rituals essential to ensuring the safe passage of the deceased into the spiritual world. Failure to perform these rituals was believed to disrupt the transition and potentially affect the living. These findings align with the literature, indicating that cultural frameworks significantly influence how individuals interpret death and cope with loss (Appel, 2011). Additionally, African Indigenous beliefs emphasize communication with ancestors, reinforcing the idea of ongoing spiritual connection (Zanardi, 2015). Africans believe that death completes an elaborate life cycle, as demonstrated in the ensuing extract:

Maybe, let's say a person has an accident. When a person has an accident in our rituals, we will go there, and we decide we go with the Sangoma [i.e. traditional healer] … to fetch his spirit so that it cannot lie there … where the accident happened. (Participant 10)

Death in the African traditional belief system is also understood as a rite of passage that allows the person’s spirit to travel on to the next world away from the physicality of this life (Obamwonyi, 2016; Rugonye & Bukaliya, 2016), which also denotes an afterlife. However, this passage to the afterlife occurs only when proper rituals and rites are conducted and the person has received a proper funeral. This point is clarified in the following excerpt:

… On the time of day of the funeral, [the] dead body is not allowed to come into the house. It must stay outside because of the accident because of the site they say in our ritual if the person goes inside the house, it means that person will call another person to die in an accident. (Participant 10)

Since rituals facilitate the rites of passage to the next world, not completing such rituals may block a person’s entry into the afterlife. Failure to observe these rites can lead to the deceased returning to trouble the surviving and living family members (Rugonye & Bukaliya, 2016). Many traditional South Africans combine traditional African beliefs with Christianity. One participant, who identified as a Christian, explained his beliefs about death and dying and the role of the ‘Sangoma’ (traditional healer), noting:

When I talk to my ancestors, I tell them that please make my dreams to be clear in such a way that I understand them, because sometimes they come in riddles and like it's very difficult to understand riddles…. Now they don't visit you in the flesh because they are not living. They are not in flesh, but they in a spirit… So, with death, that's what I believe I believe that we take the flesh and build somewhere, but the spirit will still live on... Like with the gift that I have of my calling, because if I learn, I learn different things every day, I think that's the excitement that I get with both beliefs. (Participant 9)

Furthermore, it is an African cultural belief that being in the world of the dead confers supernatural powers over those in the world of the living, such as the ability to bless or curse, and to give life or take life, among others. African Indigenous beliefs entail “patla” (speaking, praying, talking) to the ancestors and communicating with them is seen as a “calling” (Zanardi, 2015). The African cultural perspective on death holds that the deceased continues to exist in another realm, and that individuals with a specific calling can establish communication with ancestors to aid the living. This theme illustrates how interpretations and cultural practices can lead to varying levels of belief about life and death in religious traditions. Religious and cultural beliefs significantly shape individuals’ perspectives on life, death, and the afterlife. Various traditions perceive death as a transition to an afterlife, providing insights for healthcare contexts.

### Impact of Government Restrictions, Loss, Grief, and Death During COVID-19

Participants described significant disruptions caused by lockdown measures, consistent with Kübler-Ross’s model of emotional responses to loss and change (Kübler-Ross, 1969). Initial reactions included shock, denial, and uncertainty. While some participants experienced initial relief or adjustment benefits, many reported financial hardship, social isolation, and emotional distress (Duby et al., 2022; Gautam et al., 2024; Koopman, 2021).

The concept of “shadow loss” (Imperi, 2008) was evident, as participants experienced non-death-related losses such as routine, social connection, and stability. This superordinate theme relates to the participants’ journey, using Kübler-Ross’s model of loss, grief, and death, and exploring the impact of the COVID-19 pandemic on changes in work, home, family, and personal matters, as well as responses to the lockdown. The Kübler-Ross model is used in life stages of change management and in perspectives on loss, grief, and death. The theory provides valuable insights into how people respond to change. It shows how personal performance, energy, and mood vary through the normal process of human change.

Analysis of participants’ perceptions and experiences on the impact of COVID-19 generated the following subthemes: (1) Impact of the suddenness of the restrictions; (2) Lockdown and its implications; and (3) Loss, grief, and death during the pandemic.

### Impact of the Suddenness of the Restrictions

The suddenness of COVID-19 restrictions in South Africa had a profound impact. As the pandemic emerged abruptly, many individuals were caught off guard and initially unaware of its seriousness. The sudden measures, including lockdowns, business closures, and restrictions on gatherings, disrupted daily life and caused uncertainty during the initial phase. The following excerpt demonstrates this point: “…I didn't know what was happening because we were just told not to come to work” (Participant 7). Similar sentiments were expressed by another participant who noted, “… In the beginning, before lockdown, I didn't think it was that big of a deal because it was like in another country and I've never experienced a pandemic, ever” (Participant 4). This alludes to an initial sense of denial as proposed by Kübler-Ross, while the same participant, however, also stated that despite the initial lack of knowledge and unfamiliarity, once more information was obtained, fear rose, and shock set in: “… I thought it wasn't going to impact me, but, watching the news, … there was a hard lockdown, and it was a huge shock to my system” (Participant 4).

This initial sense of the unexpected announcement of the COVID-19 pandemic, shared by participants, seems to illustrate the uncertainty of the changes experienced in the early stages of the pandemic and the immense shock and fear participants faced during this time. As the stages of change unfold, the Kübler-Ross model advises that whenever someone hears bad news, the instinctive reactions are denial, uncertainty, shock, or fear. Ultimately, the initial stage is the most challenging, and overcoming denial, uncertainty, shock, or fear appears to be a challenge most people face in an overwhelming situation such as the COVID-19 pandemic. Thus, the unexpectedness of the lockdown had various implications for participants, leading to increased levels of worry and stress.

### Lockdown and its Implications

Initially, when lockdown measures were implemented in South Africa, many participants welcomed them. One participant noted: “… I felt like I needed [it]. You know, like a saving grace for the lockdown because I was not emotionally well, like a week before the COVID, the COVID lockdown” (Participant 9). Most participants enjoyed spending quality time with their family and in their homes with close ones, children, and partners, while also working from home, stating:

… At first, it was fun. We were like in a bit of a holiday. …we watched movies, we had food, and we ate with family… I think that initial time at the beginning of COVID when we had to be permanently with our son… I think that that beginning part was almost fun because it was something new,” with Participant 7 stating this as part of their reflection on early lockdown experiences. (Participant 7)

The pandemic and lockdown measures brought with it uncertainty, altered daily routines, financial pressures, and social isolation. These kinds of losses are what Imperil (2021) refers to as shadow losses, which also lead to grief. Such losses are outlined in the following quotes:

Yeah, that was the bad thing. Bad things have happened to us because many people lost their lives, many people lost their jobs and many people, they have lost their houses, then they had to adapt. How many people now are on the unemployment rate (sic)? There are a lot, and people are still struggling even now, even if we can, we can say we are going to recover we are going to take more years to recover. COVID has made us live like this, how many people now, are still looking for jobs and all that, and companies have closed. (Participant 1)

… It's hard because you don't know what everyone else is going through, and it's just horrible to hear, you know, that one or two have been successful during COVID. There were one or two uh suicides which, like you, think, like why, you know, and young people, and then now we [go] back to work. Other pressures are extra because you [are] going to make up for the money. We lost during COVID… (Participant 2).

This indicates that COVID-19 had huge financial implications, which aligns with reports of over a million South Africans losing their jobs due to the initial five-month-long lockdown (Koopman, 2021). Feelings of isolation and loneliness rose during the pandemic and restrictions, and some participants found social isolation very difficult. These findings are supported by studies in South Africa and abroad. In a study conducted by Duby et al. (2022) in South Africa, it was found that COVID-19 restrictions on movement, continuous lockdown, and forced quarantine exacerbated stress and anxiety, with participants expressing emotions ranging from fear, despair, frustration, boredom, and isolation to loneliness. Gautam et al. (2024) emphasizes the importance of recognizing diverse coping methods for promoting mental well-being during challenging times for everyone in society. Additionally, Zhang et al. (2021) explored religious and spiritual struggles and coping strategies during the pandemic, with participants describing various struggles, including interpersonal, moral, doubt, and ultimate meaning struggles. The study will expand on the impact of loss, grief, and death during this challenging time.

### Loss, Grief, and Death During the Pandemic

Participants experienced multiple forms of loss, including bereavement, loss of routine, and reduced social interaction. The inability to perform traditional mourning rituals contributed to prolonged and complicated grief. Participants’ experiences align with research on grief progression, although the process was often non-linear (Kübler-Ross, 1969). Emotional responses included sadness, anger, and eventual acceptance, highlighting the complexity of grief during the pandemic. This is emphasized in the following excerpt:

I think it's important for us to acknowledge all kinds of losses and not just focus on the big ones like the, you know, death or the end of a marriage or in the friendship and that kind of thing (Participant 7).

This indicates that loss during COVID-19 was not just perceived in terms of death, but also concerning any kind of loss experienced. The same participant elaborated this notion of loss as being all-inclusive, but at the same time associated loss with a divine decree:

Loss means that something is taken away that you were used to, or someone taken away. This [is] in your circle, so when it's snatched away, it forces you to change your perception or your reality or day-to-day. For me, I perceive loss as everything happens for a reason, and when we refer to loss of life; I know there's a time for everyone, so as much as you may want to … plan life, I feel everybody is being tested anyway, so if it's a loss of life, especially the closer to you, it does take time for us as humans, to get used to the loss, but I don't feel it was unclear or forced on [us]. I see it was their time, and we need to make peace with that. Then there’s a loss of a job or loss of something else, such as a home, a relationship, or a sense of purpose. (Participant 7).

The experience of loss was very common among participants, and all had faced some form of loss on their journey during COVID-19. For some participants, loss was experienced as loss of a loved one, but also as loss of completing final rites and rituals for the deceased. One participant noted: “My uncle, my mother's brother, so I lost him. I didn’t even get to go to the funeral, which is very sad and [un]just. I just thought it wouldn't end (Participant 4)”. Another participant indicated: “Traditionally, people suffered as they could not do their rituals, and it is a big part of their culture and who they are. They could not sacrifice when someone died and feed the community. They were lost” (Participant 10).

Loss and grieving were experienced in multiple ways by participants, and the stages of grief as espoused by Kübler-Ross manifested differently. Participants progressed through the different stages of grieving (Denial, Anger, Bargaining, Depression, Acceptance) while coping with COVID-19’s impact and other challenges at the time. One participant noted that during COVID, she “was so distraught … heartbroken… very sad, … would just sit … just start crying at some point” (Participant 7). She further acknowledged that she “was in a depressive phase … [and] was really angry for a long time…” (Participant 7). As noted by Kübler-Ross (1996), the participant experienced and expressed anger, directed at close family members (Participant 7). She experienced depression and depressive symptoms, including sadness, crying, distress, panic, an elevated heart rate, and shakiness, and she tried to avoid thinking about her loss, and then came acceptance. Understanding how these stages may work differently, not necessarily in a linear manner, regardless of the type of loss, may guide practitioners in supporting patients optimally.

## Spiritual and Religious Coping Mechanisms Used to Navigate Loss, Death, and Grief

Death, grief, negativity, and maladaptive life experiences (such as adjusting to COVID-19 restrictions and pandemic ramifications) are unavoidable aspects of life, and overcoming these challenges, obstacles, and adversities constitutes a significant pursuit (Kgatle & Segalo, 2021). Considering the unique circumstances and restrictions imposed due to COVID-19, dealing with loss, death, and grieving was extraordinarily difficult for many people. Recognizing coping methods and the role of spirituality is crucial during challenging times, especially amidst the pandemic and future crises. Participants’ experiences were multifaceted, not confined to single events. This theme unpacks the coping mechanisms participants used to deal with the various challenges related to loss, death, dying, and grief during the pandemic. Spirituality and religion were central coping mechanisms for participants. Religious practices such as prayer, rituals, and faith-based meaning-making supported emotional resilience during the pandemic. These findings align with literature highlighting the role of spirituality in coping with adversity (Kgatle & Segalo, 2021; Safdar et al., 2023). Religion provided individuals with a framework for interpreting suffering and finding meaning in difficult circumstances.

Spiritual coping also facilitated emotional regulation and psychological well-being (Krok, 2015). Participants’ experiences underline the importance of incorporating spiritual resources into mental health interventions. Participants in this study explained how spirituality and their religious affiliation, in many ways, helped them get through uncertain times during the pandemic, when the country and the world were still unsure about the pandemic's magnitude. One participant noted the role prayer plays in his life:

I pray to God and even if there are delays, He will come through. There is a quote in the Bible for everything, and I go to church every week. At our church, every day of the week you can pray for something, relationships, finances, … health. We are children of God… You see traditionally people use trees, plants, and nature to heal and make remedies when they are sick or feel stuck in some area of life… Like when you are angry or you are too sad, or you cry all the time. You need to pray, allow yourself to feel those emotions and trust God as a Christian (Participant 10).

Safdar et al. (2023) underscore the role of religion and spirituality as coping mechanisms during the pandemic, confirming that rituals and religious practices aid in dealing with loss and death. Another participant indicated that her faith deepened during the pandemic and that her relationship with the divine grew. She turned more to Islam as her source of support and followed its practices.

And if we stick to that often, you'll be grounded ... Yeah, it's such a powerful tool that it is one of the simplest things… in Islam. So, I find that in religion, there are a lot of things that help me. Not only through the pandemic but through life, if we use it the way it's meant to be used. And then if we [are] stuck in life, we've reverted to the Quran [i.e. Muslim scripture], where whatever guidance you need, whether it's finance, whether it's whatever, and at some point, where you need to surrender your problems to God alone (Participant 7).

A study by Krok (2015) found a connection between religious coping and psychological wellbeing, highlighting that individuals turn to religious beliefs and practices to seek meaning in complicated, difficult, and incomprehensible occurrences. Religiosity and spirituality significantly influence recovery from physiological and psychological COVID-19 challenges for many participants across various spiritual and religious spectrums. As demonstrated in the ensuing quote, one participant noted that during COVID19, she just resorted to prayer:

So, I'm Hindu. I think at the time, I didn't realize that it happened or anything, but I realized that I started praying a lot more and my faith did become stronger. It was probably because I just had space and my brain was overworking. So, I think that would have helped my spiritual beliefs. I’m not very religious, but I'm spiritual if that makes sense (Participant 8).

Participants turned to their faith and spiritual practices to deal with loss and death during and in the immediate aftermath of the pandemic, even though new and adaptive technologies, such as online platforms, seemed to cope better. The following quotes illustrate this point:

… I align [to] me to religion a lot, I prayed daily because, like, even on hard days like you, you won't expect like a small win or something good to happen or something that you thought would be hard turned out to be easier to manage. (Participant 4)

…I believe in God that God is there. So how I do things is that before anything else I pray first, which I pray to God and then after praying, that's when I will, “Patla,” when I “Patla,” it's like speaking to my ancestors and with my ancestors. It's like in English; I can call them angels. To me they are angels. (Participant 9)

In their spiritual and religious journeys, people can take several pathways toward significant destinations in their lives. At their core, both religion and spirituality involve dynamic, exploratory processes centred on the sacred (Harper & Pargament, 2015). Spirituality and religion are multidimensional and multilevel processes. These paths are not necessarily followed in isolation from other people. So, the meanings of religion and spirituality continue to evolve.

## Limitations

The study’s limitations include a small, homogeneous sample, which limits the generalizability of the findings. Results primarily reflect South Africans, mainly from health, financial, and educational professions, and cannot be broadly applied to the entire population. Participants’ views on loss, grief, and death were subjective and unique to each individual. This study forms part of a broader master’s study and, due to the word/page limit, the authors were restricted in their selection of quotes.

## Recommendations

Based on the findings of this study, several recommendations are proposed to enhance mental health support and understanding during times of crisis, particularly through spiritual and religious coping mechanisms. Coping with mental health challenges is a complex and ongoing process. The COVID-19 pandemic significantly increased global levels of depression, anxiety, stress, insomnia, denial, fear, and anger (Torales et al., 2020). While some of these challenges have persisted, others have evolved as restrictions have eased. Participants emphasized the need to raise awareness and reduce misconceptions about mental health, noting that stigma continues to affect workplace dynamics and personal relationships. Improving mental health literacy—including understanding mental health disorders, risk factors, and available support—is essential for effective coping. This study highlights the importance of spiritual and religious resources in managing grief, loss, and death, and underscores the value of seeking support and adopting meaningful coping strategies.

Future research should involve larger and more diverse samples to improve generalizability, reduce bias, and detect nuanced effects. Combining qualitative and quantitative methods is also recommended to provide a more comprehensive understanding of how spirituality and religion contribute to healing during crises.

## Conclusion

This study explored the role of spirituality and religion in coping with the psychological impact of loss, grief, and death during the COVID-19 pandemic (Centers for Disease Control and Prevention, 2021). Findings revealed that spiritual and religious beliefs played a central role in helping participants navigate these profound life transitions, though many practices were adapted in response to pandemic-related restrictions. Faith-based strategies—such as prayer, meditation, and religious rituals—emerged as key coping mechanisms, offering emotional support and a sense of continuity during a time of uncertainty (National Alliance on Mental Illness, 2016).

While the study mentioned broader coping strategies such as social support, exercise, and professional help, it primarily focused on spiritual and religious responses. Therefore, the emphasis in this conclusion remains on those domains (BusinessTech, 2020; Lim, 2022; Newton & McIntosh, 2013; Sampaio et al., 2022; Smith et al., 2024). Importantly, the study highlights how spiritual and religious convictions shape individuals’ understanding of mortality, offering frameworks for meaning making and connection. These beliefs often interpret death not as an end, but as a transition to another form of existence—an afterlife—challenging purely biomedical views of death.

Loss, grief, and mortality are transformative experiences that reshape our psychological, social, and spiritual landscapes. As individuals confront these realities, they often re-evaluate their values, strengthen social bonds, and adjust their life paths. Understanding these diverse perspectives is essential for holistic support during times of crisis.

## Data Availability

The data presented in this study are available on request from the corresponding author(s).
